# Economic evaluation of the sFlt-1/PlGF ratio for the short-term prediction of preeclampsia in a Japanese cohort of the PROGNOSIS Asia study

**DOI:** 10.1038/s41440-021-00624-2

**Published:** 2021-02-16

**Authors:** Akihide Ohkuchi, Hisashi Masuyama, Tatsuo Yamamoto, Takashi Kikuchi, Naoko Taguchi, Cyrill Wolf, Shigeru Saito

**Affiliations:** 1 Jichi Medical University Hospital, Tochigi, Japan; 2 Okayama University, Okayama, Japan; 3 Nihon University Itabashi Hospital, Tokyo, Japan; 4 Roche Diagnostics K.K., Tokyo, Japan; 5 Roche Diagnostics International Ltd, Rotkreuz, Switzerland; 6 Toyama University Hospital, Toyama, Japan

**Keywords:** Cost saving, Prediction, Japan, Preeclampsia, sFlt-1/PlGF ratio

## Abstract

The PRediction of short-term Outcomes in preGNant wOmen with Suspected preeclampsIa Study (PROGNOSIS) Asia validated the use of the soluble fms-like tyrosine 1/placental growth factor (sFlt-1/PlGF) ratio cutoff value of ≤38 to rule out the occurrence of preeclampsia in the short term in Asian women. We assessed the economic impact of the introduction of the sFlt-1/PlGF ratio test for predicting preeclampsia in Japan using data from the Japanese cohort of PROGNOSIS Asia. The cost analysis was developed with estimates in either a no-test scenario, with clinical decisions based on standard diagnostic procedures alone, or a test scenario, in which the sFlt-1/PlGF ratio test was used in addition to standard diagnostic procedures. For both scenarios, rates of hospitalization and other test characteristics were obtained from the results for the Japanese cohort in PROGNOSIS Asia. The total cost per patient was the main outcome of this cost analysis model. Introduction of the sFlt-1/PlGF ratio test using a cutoff value of 38 resulted in a reduced hospitalization rate compared with the rate in the no-test scenario (14.4% versus 8.7%). The reduction in the rate of hospitalizations led to an estimated 16 373 JPY reduction in healthcare costs per patient. The sFlt-1/PlGF ratio test is likely to reduce the unnecessary hospitalization of women at low risk of developing preeclampsia in the short term while also identifying high-risk individuals requiring appropriate management. Reducing unnecessary hospitalizations would result in significant cost savings in the Japanese healthcare system.

## Introduction

Preeclampsia is a hypertensive disorder of pregnancy (HDP), defined as new-onset hypertension and proteinuria or other maternal organ dysfunction after 20 weeks of gestation [1]. Globally, preeclampsia affects 2–8% of pregnancies [2, 3] and ~3% of singleton pregnancies in Japan [4]. It is associated with substantial maternal morbidity and mortality [5, 6]. In Japan, between 1989 and 2004, 193 cases of maternal deaths were due to direct obstetric causes; of those deaths, 41 (21.2%) were associated with disseminated intravascular coagulation due to HDP [7]. Preeclampsia also contributes significantly to healthcare costs [5, 8, 9].

Guidelines from the Japan Society of Obstetrics and Gynecology recommend the hospitalization of women diagnosed with preeclampsia but do not provide clear guidance on the management of women with risk factors only for the condition [10]. There is no centralized maternal care system in Japan, and the use of midwives is uncommon. Therefore, women often visit obstetricians in the clinic, which may further impact the management of women with risk factors for preeclampsia. As such, there is an unmet clinical need for objective indicators to predict preeclampsia. Currently, women are referred to perinatal care centers only if they develop severe preeclampsia, hemolysis, elevated liver enzymes, low platelet count (HELLP) syndrome, seizures, or mild/moderate/accidental hypertension.

Preeclampsia is associated with dysregulation of angiogenic factors such as soluble fms-like tyrosine 1 (sFlt-1) and placental growth factor (PlGF) [11–14]. Quantification of the sFlt-1/PlGF ratio, derived from Elecsys® sFlt-1 and Elecsys PlGF immunoassay (Roche Diagnostics, Mannheim, Germany) results, has been shown to be a useful biomarker test for aiding the diagnosis and short-term prediction of preeclampsia [15–22].

The global, multicenter, noninterventional PRediction of short-term Outcomes in preGNant wOmen with Suspected preeclampsIa Study (PROGNOSIS) derived and validated an sFlt-1/PIGF ratio cutoff of 38 to predict the development of preeclampsia in women with clinical suspicion of the condition. The negative predictive value (NPV) of an sFlt-1/PlGF ratio ≤38 to rule out preeclampsia was 99.3% within 1 week and 94.3% within 4 weeks; the positive predictive value (PPV) to rule in preeclampsia within 4 weeks was 36.7% [17, 22, 23]. PROGNOSIS Asia validated the sFlt-1/PlGF ratio cutoff of 38 for short-term preeclampsia prediction in Asian women with suspected preeclampsia [15]. Analysis of PROGNOSIS Asia data demonstrated an NPV of 98.6% for an sFlt-1/PlGF ratio of ≤38 to rule out preeclampsia within 1 week and an NPV of 95.1% to rule it out within 4 weeks. The PPV to rule in preeclampsia within 1 week was 17.9%, and the PPV to rule it in within 4 weeks was 30.3%.

The economic impact of implementing the sFlt-1/PlGF ratio test, based on PROGNOSIS data, has been assessed in the UK, Italy, Brazil, Germany, Switzerland, and the US [24–30]. The authors concluded that introducing the test into clinical practice for women with suspected preeclampsia would provide cost savings for the respective healthcare providers, mainly due to the increased ability to rule out preeclampsia and thereby reduce unnecessary hospitalizations. However, this type of economic assessment is specific to the individual patient population and healthcare provider tested and cannot be extrapolated to other healthcare systems. To date, the economic impact of this test has not been assessed using patient-level data from PROGNOSIS Asia. The present analysis thus aimed to evaluate the economic impact of implementing the sFlt-1/PlGF ratio test for the short-term prediction of preeclampsia in Japanese women with suspected preeclampsia using patient-level data from PROGNOSIS Asia [15, 31].

## Methods

### The Japanese cohort from PROGNOSIS Asia

PROGNOSIS Asia was a prospective, multicenter observational study of 764 women with suspected preeclampsia who were enrolled at 25 sites across Asia between December 2014 and December 2016 [15]. The Japanese cohort in PROGNOSIS Asia included 192 enrolled women at eight sites in Japan between June 2015 and May 2016. The key inclusion criteria for the Japanese cohort were being a pregnant woman ≥18 years of age who presented with suspected preeclampsia from 18 weeks + 0 days gestation to 36 weeks + 6 days gestation. The inclusion criteria were adapted to align with local practice guidelines as required; the criterion relating to the lower limit of blood pressure was adapted to reflect Japanese practice, and the lower limit of the eligible gestational age range was adapted to 18 weeks (rather than 20 weeks for the other Asian countries). The key exclusion criteria were manifest preeclampsia or confirmed diagnosis of HELLP syndrome; multiple pregnancies; confirmed diagnosis of a fetal chromosomal abnormality; or treatment with an investigational medicine within 90 days. The study protocol was approved by local ethics committees and institutional review boards at each of the eight Japanese sites involved prior to study initiation, and all participants provided written informed consent. The study was conducted in accordance with the Declaration of Helsinki and International Conference on Harmonisation guidelines for Good Clinical Practice.

Patient-level data from the Japanese cohort in PROGNOSIS Asia were used to determine the following factors in the present economic model: (a) the proportion of women hospitalized with suspected preeclampsia in current practice (i.e., in the absence of sFlt-1/PlGF ratio results); (b) the correlation between hospitalization and the sFlt-1/PlGF ratio (where values ≤38 suggest low risk and >38 suggest high risk [22]); and (c) the relationship among the sFlt-1/PlGF ratio, hospitalization, and a confirmed diagnosis of preeclampsia. The study was also used to provide information on the length of hospital stay before and after preeclampsia onset and for women who did not develop preeclampsia.

### Cost analysis model

For this cost analysis, an economic model was developed to estimate direct medical costs associated with the diagnosis and management of women with suspected preeclampsia from a Japanese healthcare system perspective. The model simulated the progression of a cohort of Japanese women through a treatment pathway from the first presentation with clinical suspicion of preeclampsia to the point of delivery, which was determined by the assessed risk of developing preeclampsia and the consequent decision of whether to hospitalize the patient or manage in an outpatient setting. The model also included the cost of neonatal intensive care unit (NICU) admission and treatment of neonates with respiratory distress syndrome (RDS) born to mothers with suspected preeclampsia. The model assumed that every neonate born with RDS would be admitted to the NICU; that the rate of RDS in neonates from hospitalized mothers with suspected preeclampsia is 12%; and that hospitalizing women with suspected preeclampsia based on the sFlt-1/PlGF ratio result and corticosteroid administration, would reduce the RDS rate by 20% [32]. The calculation of a 20% reduction was derived from PreOS, a multicenter, noninterventional study run in Germany and Austria evaluating the sFlt-1/PlGF ratio test for its use in diagnosis and clinical decision making [33]. The study report showed that the overall reduction in the RDS rate in PreOS was 25.6% [unpublished data]; however, the sFlt-1/PlGF ratio cutoff utilized to rule out preeclampsia in PreOS was 33, whereas a higher cutoff of 38 was used in PROGNOSIS Asia. Therefore, we assumed that the reduction in the RDS rate would decrease from 25.6 to 20%.

The model assumptions were estimated based on values from the Neonatal Research Network of Japan database [32]. The incremental value of implementing the sFlt-1/PlGF ratio test was evaluated by comparing expected management costs in two scenarios. In the no-test scenario, diagnosis and management of women with suspected preeclampsia were performed according to current standard-of-care procedures; the hospitalization rate was based on the observed rate in the Japanese cohort in PROGNOSIS Asia. In the test scenario, diagnosis and management of women with suspected preeclampsia were performed according to current standard-of-care procedures plus the results of the sFlt-1/PlGF ratio test. The results of the sFlt-1/PlGF ratio test enabled classification of the women as either low (≤38) or high (>38) risk for developing preeclampsia. The proportion of women in each group was based on the observed proportions in the Japanese cohort in PROGNOSIS Asia. Notably, in PROGNOSIS Asia, the results of the sFlt-1/PlGF ratio test were blinded to the clinicians to ensure that clinical decision making was not impacted by the results. In the low-risk group, the hospitalization rate (0.56%) was based on the assumption that women with an sFlt-1/PlGF ratio ≤38 (with an NPV to rule out PE within 1 week of 98.6% and a value of 95.1% to rule out within 4 weeks [15]) would be hospitalized if their blood pressure was ≥160/110 mmHg (as per the Best Practice Guide 2015 for Care and Treatment of Hypertension in Pregnancy [34]). In the high-risk group, the model used a conservative estimate for the hospitalization rate (40%) based on the observed rate in the Japanese cohort in PROGNOSIS Asia, where the hospitalization rate was 27%.

The patient pathway was based on the PROGNOSIS Asia study and adapted to Japanese patient management procedures; the model assumed that patients not admitted to the hospital were managed in an outpatient setting. The incidence of preeclampsia was assumed to be unaffected by the introduction of the sFlt-1/PlGF ratio test. No direct information was available from the PROGNOSIS study to determine what clinical decisions might have been made if physicians had been given access to sFlt-1/PlGF ratio results. A clinical algorithm was developed to estimate the disposition of women according to their sFlt-1/PlGF ratio.

### The model cohort

A model cohort of 31,000 women (based on estimates of pregnant women with preeclampsia in Japan at any given time) with suspected preeclampsia and the Japanese-specific cohort from PROGNOSIS Asia were used to compare the no-test and test scenarios.

### Costs used in the analysis

The expected costs of caring for women with suspected preeclampsia and neonates with RDS used in the modeling were based on annual data from the Ministry of Health, Labor & Welfare report (Table 1). The model assumes that all patients treated with high-intensity management are prescribed oral corticosteroids to reduce the risk of complications at a cost of 1752 Japanese yen (JPY) per patient. The cost of the sFlt-1/PlGF ratio test was estimated to be 9000 JPY. Outpatient costs were calculated for every woman in the model, including hospitalized women, who were assumed to be treated in an outpatient setting at some stage.

**Table 1:** Expected maternal and neonatal care costs used in the economic modelling (based on annual data from Ministry of Health, Labour & Welfare reports [37–39])

Cost type	Cost, JPY
Maternal care	
Outpatient^a^	57,053
Hospitalization (per day)	87,300
sFlt-1/PlGF ratio test	9000
Neonatal care	
Maternal corticosteroid treatment	1752
NICU stay (per day)	101,302

*JPY* Japanese yen, *NICU* neonatal intensive care unit, *PlGF* placental growth factor, *sFlt-1* soluble fms-like tyrosine kinase-1

^a^Outpatient costs were calculated for every woman entering the model, including those who were hospitalized, as it was assumed that hospitalized women would also be treated in an outpatient setting at some stage

### Sensitivity analyses

Sensitivity analyses were conducted to test the robustness of our results for a range of scenarios in which key model parameters were altered. The analysis included (a) an increase in the hospitalization rate in the no-test scenario to the overall hospitalization rate in PROGNOSIS Asia; (b) variations in the cost of the sFlt-1/PlGF ratio test, whereby the cost was increased and reduced by 20%; (c) a second sFlt-1/PlGF ratio test for every woman (also an estimated cost of 9000 JPY); (d) an increase in the hospitalization rate for women with sFlt-1/PlGF ratios ≤38 to 4%; (e) a decrease in the hospitalization rate for women with sFlt-1/PlGF ratios ≤38–0%; and (f) an increase of 10 percentage points in the hospitalization rate for women with sFlt-1/PlGF ratios >38.

## Results

### Preeclampsia-related hospitalization rates

**Fig. 1:**
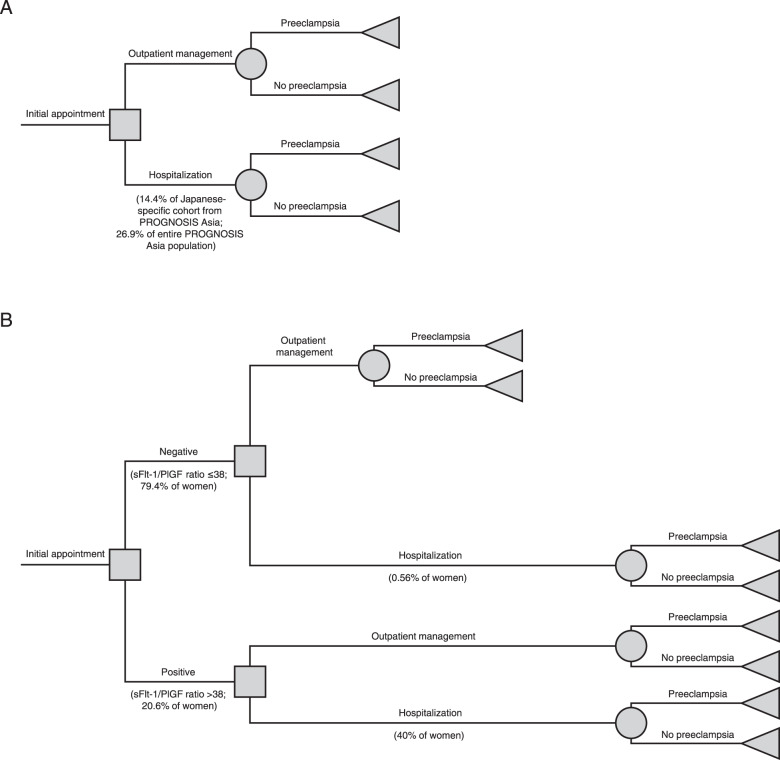
Decision trees in the (**A**) no-test scenario (i.e., current standard of care) and (**B**) test scenario (i.e., current standard of care plus the sFlt-1/PlGF ratio test). *PlGF*, placental growth factor; *PROGNOSIS*, PRediction of short-term Outcomes in preGNant wOmen with Suspected preeclampsIa Study; *sFlt-1*, soluble fms-like tyrosine kinase-1

Data for 180 Japanese women (out of 192 women enrolled) with suspected preeclampsia from PROGNOSIS Asia were eligible for the analysis. In the no-test scenario, 14.4% of women were hospitalized, and 85.6% were treated in an outpatient setting (Fig. 1a). The hospitalization rate for this cohort was lower than the overall rate for the entire PROGNOSIS Asia population, in which 26.9% of women were hospitalized. The average length of hospital stay was 10.4 days in the no-test scenario and 10.2 days in the test scenario (Supplementary Table 1; unpublished data). The overall number of inpatient days included women admitted before preeclampsia (average 4 days); the length of stay for those admitted for the birth period varied depending on whether the women developed preeclampsia (13.6 days) or not (9.3 days). A larger number of women were admitted before the onset of preeclampsia in the no-test scenario (*n* = 4476) than in the test scenario (*n* = 2691). This model assumes that the incidence of preeclampsia is independent of sFlt-1/PlGF ratio testing; as such, the number of birth episodes both with and without preeclampsia remained the same between the test and no-test scenarios.

In the test scenario, 79.4% of women had sFlt-1/PlGF ratios ≤38, and 0.56% of them were hospitalized. The remainder of the women (20.6%) had sFlt-1/PlGF ratios >38, and 40% of them were hospitalized (Fig. 1b). The hospitalization rate for PROGNOSIS Asia was 27% in this cohort, but a more conservative rate of 40% was used for the present analysis. This resulted in an overall hospitalization rate of 8.7% in the test scenario.

Of the women who were hospitalized in the test scenario, 41.4% developed preeclampsia in the hospital. By contrast, the rate of women who developed preeclampsia while in the hospital in the no-test scenario was 24.1%. These results support the hypothesis that the test facilitates the appropriate identification and management (i.e., through hospital admission) of women at higher risk of developing preeclampsia, while women at lower risk may be managed in an outpatient setting.

### NICU admission rates for RDS

Applying a 12% RDS rate (for newborns from hospitalized mothers with signs and symptoms of preeclampsia) and a 20% reduction in the RDS rate (if the test is used) to a model cohort of 31,000 women (assuming that the full model cohort of women with suspected preeclampsia would have access to the test) would result in 537.17 NICU admissions in the no-test scenario and 429.73 NICU admissions in the test scenario (Supplementary Table 2).

### Base-case cost saving and sensitivity analyses

Based on the expected maternal and neonatal care costs shown in Table 1, introduction of the sFlt-1/PlGF ratio test is expected to result in cost savings of 16,373 JPY per patient compared with costs in the no-test scenario (Table 2; Supplementary Table 2). These cost savings are largely due to the reduction in hospitalization. To perform the sensitivity analyses, cost savings of 16,373 JPY per patient were used as the base case. The sensitivity analyses supported the robustness of the value of investigating the sFlt-1/PlGF ratio in reducing costs, as the cost savings across all scenarios ranged from 6782 to 69,482 JPY (Table 2; Supplementary Fig. 1).

**Table 2:** Cost savings associated with implementation of the sFlt-1/PlGF ratio in the base case and sensitivity scenario analyses

Scenario	Hospitalization rate without sFlt-1/PlGF ratio, %	Cost per sFlt-1/PlGF ratio test, JPY	sFlt-1/PlGF ratio retest option	Hospitalization rate with sFlt-1/PlGF ratio result ≤38, %	Hospitalization rate with sFlt-1/PlGF ratio result >38, %	Cost saving per patient, JPY
Base case (including NICU admission for RDS)	14.4	9000	No	0.56	40	16,373
Hospitalization rate without sFlt-1/PlGF ratio increased to overall rate in PROGNOSIS Asia	26.9	9000	No	0.56	40	69,482
sFlt-1/PlGF ratio cost per test reduced by 20%	14.4	7200	No	0.56	40	18,173
sFlt-1/PlGF ratio cost per test increased by 20%	14.4	10,800	No	0.56	40	14,573
sFlt-1/PlGF ratio retest considered for every woman	14.4	9000	Yes	0.56	40	7373
Hospitalization rate with sFlt-1/PlGF ratio ≤38 increased to 4%	14.4	9000	No	4	40	6782
Hospitalization rate with sFlt-1/PlGF ratio ≤38 reduced to 0%	14.4	9000	No	0	40	17,934
Hospitalization rate with sFlt-1/PlGF ratio >38 increased to 50%	14.4	9000	No	0.56	50	9157

*JPY* Japanese yen, *NICU* neonatal intensive care unit, *PlGF* placental growth factor, *RDS* respiratory distress syndrome, *sFlt-1* soluble fms-like tyrosine kinase-1

## Discussion

Our economic analysis of patient-level data from PROGNOSIS Asia shows that implementation of the sFlt-1/PlGF ratio as a diagnostic aid in current standard-of-care procedures could reduce the hospitalization rate for Japanese women with suspected preeclampsia from 14.4 to 8.7%. Notably, the introduction of the sFlt-1/PlGF ratio test would decrease the number of NICU admissions of neonates with RDS born to these women and the associated costs. Our findings suggest that implementation of the sFlt-1/PlGF ratio in routine clinical practice may enable better stratification of women at low-and high-risk of developing preeclampsia and thus facilitate appropriate clinical decision making. Management decisions that include the results of the sFlt-1/PlGF ratio correlated with preeclampsia outcomes better than current diagnostic procedures alone. For patients at low risk for preeclampsia, the reduction in unnecessary hospitalizations and extended monitoring likely improves the cost-effectiveness for healthcare providers in Japan.

In the base case scenario, the decreasing rates of hospitalization and NICU admission due to the introduction of the sFlt-1/PlGF ratio test were associated with cost savings of 16,373 JPY per patient compared with the costs using current standard-of-care procedures alone. The additional cost of the sFlt-1/PlGF ratio test is more than offset by a reduction in hospital resource use. The exact reduction in the hospitalization rate and the associated cost savings are dependent on local factors; however, our sensitivity analyses demonstrate that our base case cost assumptions are robust to changes in key parameters in the modeling, including running the test twice for each woman.

Assuming that there are 31,000 pregnant women with suspected preeclampsia in Japan at any given time, the budget impact of including the sFlt-1/PlGF ratio test in routine clinical practice could be 507,560,536 JPY in total savings for the Japanese healthcare system. Prior to implementation by healthcare providers, the cost-effectiveness of any new test must be demonstrated in addition to its clinical performance. Similar research on the cost analysis of introducing the sFlt-1/PlGF ratio test has been performed using population-level data from PROGNOSIS in the UK, Italy, Germany, Brazil, Switzerland, and the US [24–30]. In PROGNOSIS, the sFlt-1/PlGF ratio has been shown to improve the short-term prediction of preeclampsia in women with clinical suspicion of the condition, and NICE guidelines recommend the use of the sFlt-1/PlGF ratio test to rule out preeclampsia, in addition to standard care, in women between 20 and 34 weeks + 6 days of gestation [35]. These guidelines state that for women presenting with suspected preeclampsia at <35 weeks of gestation (base case), total costs were £6,456 when using the Elecsys sFlt-1/PlGF ratio test, compared with £8,945 for the standard of care, yielding cost savings of £2,488 per patient. However, it must be considered that NICE guidelines are based on the UK healthcare system and thus cannot directly inform or be used as a comparator for clinical practice in Japan. A cutoff value of ≤38 has a high NPV of 99.3% for ruling out the onset of preeclampsia within 1 week [17, 22]. Analysis of the economic impact in the UK indicated that hospitalizations of women with suspected preeclampsia were reduced by 56%, resulting in net savings of £344 per patient for the healthcare provider [28]. The substantial cost saving results per patient were replicated in Italy (€670) [25], Germany (€361) [27], Brazil (R$185–636) [24], Switzerland (€346) [26], and the US ($1,215) [29]. Taken together, these studies show that the sFlt-1/PlGF ratio test yields cost savings for different populations/geographical areas and from the perspective of a range of healthcare providers with different procedures and financial models.

A strength of the present analysis is that the economic model used real-world, patient-level data collected from a large observational study (PROGNOSIS Asia) [15]. From these data, we were able to extract data from the Japanese cohort. Thus, our results are highly likely to be representative of current clinical practice for managing preeclampsia in Japan.

A further strength of the present analysis is that our modeling included the cost of neonatal care (i.e., NICU admission and corticosteroid treatment) for neonates with RDS born to mothers with preeclampsia. Previous research has suggested that, in the context of preeclampsia, the cost burden for neonatal care may exceed that for maternal care in the first 12 months after delivery [9]. Furthermore, research has shown that the use of antenatal corticosteroids may reduce the costs of medical services and the length of hospitalization for preterm infants [36]. The inclusion of these costs in our model ensures that the cost-saving results of our study provide an accurate representation of the burden of preeclampsia on the Japanese healthcare system. A range of sensitivity analyses were performed to check the robustness of our results to changes in key model parameters.

This study has limitations. It is conceded that a randomized interventional study on the actual economic impact of using the sFlt-1/PlGF ratio to rule out preeclampsia could further strengthen our findings by quantifying the value of the test more accurately in routine clinical practice. Moreover, incorporating additional data on delivery and birth outcome parameters (e.g., length of pregnancy, cesarean section rate, birth weight) into the test and no-test scenarios could further inform this model; however, these data could not be included in the present study due to a lack of available data. Furthermore, this model assumes that all women treated with high-intensity management are prescribed oral corticosteroids to reduce the risk of complications; however, Japanese guidelines recommend that corticosteroids are administered by intramuscular injection rather than administered orally [34]. Therefore, the cost savings in real-world practice may differ slightly from those presented in the present model.

## Conclusions

Our economic analysis demonstrates that implementing the sFlt-1/PlGF ratio test for the management of women with suspected preeclampsia in Japan may reduce unnecessary hospitalizations. Furthermore, reducing unnecessary hospitalizations is associated with cost savings for Japanese healthcare providers.

## Supplementary information

Supplementary Table 1

Supplementary Table 2

Supplementary Figure 1
