# Supplementary material for: Economic evaluation of the sFlt-1/PlGF ratio for the short-term prediction of preeclampsia in a Japanese cohort of the PROGNOSIS Asia study
Source: Hypertens Res. 2021 Feb 16;44(7):822–9. doi: 10.1038/s41440-021-00624-2 (PMC8255211; doi:10.1038/s41440-021-00624-2)
Supplement: Supplementary file 1 — Supplementary Table 1 [file 41440_2021_624_MOESM1_ESM.docx]

**Supplementary Table 1** Average length of stay data from PROGNOSIS Asia (Japanese specific cohort)

|  | No-test scenario | | | Test scenario | | |
| --- | --- | --- | --- | --- | --- | --- |
|  | Number of women | Average length of stay (days) | Total inpatient bed-days | Number of women | Average length of stay (days) | Total inpatient bed-days |
| Total at risk | 31 000 | 10.4 | 323 624 | 31 000 | 10.2 | 316 502 |
| Hospitalized pre-preeclampsia | 4476.4 | 4.0 | 17 905 | 2691.4 | 4.0 | 10 765 |
| Birth episode with preeclampsia | 4136.9 | 13.6 | 56 179 | 4136.9 | 13.6 | 56 179 |
| Birth episode without preeclampsia | 26 863.1 | 9.3 | 249 558 | 26 863.1 | 9.3 | 249 558 |

*PROGNOSIS*, prediction of short-term outcome in pregnant women with suspected preeclampsIa study.
