# Supplementary material for: Economic evaluation of the sFlt-1/PlGF ratio for the short-term prediction of preeclampsia in a Japanese cohort of the PROGNOSIS Asia study
Source: Hypertens Res. 2021 Feb 16;44(7):822–9. doi: 10.1038/s41440-021-00624-2 (PMC8255211; doi:10.1038/s41440-021-00624-2)
Supplement: Supplementary file 2 — Supplementary Table 2 [file 41440_2021_624_MOESM2_ESM.docx]

**Supplementary Table 2** Hospitalization and treatment costs for a patient cohort of 31 000 pregnant women with suspected preeclampsia, in the no test and test scenarios

|  | No test scenario | | Test scenario | | Difference | |
| --- | --- | --- | --- | --- | --- | --- |
|  | Cases | JPY | Cases | JPY | Cases | JPY |
| Outpatient costs* |  | 1 768 643 000 |  | 1 768 643 000 |  | 0 |
| Test cost |  | 0 |  | 279 000 000 |  | 279 000 000 |
| Neonatal intensive care |  | 792 299 766 |  | 633 839 813 |  | -158 459 953 |
| Number of neonates admitted to intensive care | 537.17 |  | 429.73 |  | -107.43 |  |
| Hospitalization |  | 28 254 026 771 |  | 27 629 053 547 |  | -624 973 224 |
| Number of women hospitalized | 4476.40 |  | 2691.38 |  | -1785.02 |  |
| Corticosteroids |  | 7 842 653 |  | 4 715 294 |  | 3 127 359 |
| Total costs per cohort |  | 30 822 812 190 |  | 30 315 251 654 |  | -507 560 536 |
| Total costs per patient |  | 994 284 |  | 977 911 |  | -16 373 |

*Outpatient costs were calculated for every woman entering the model, including those who were hospitalized, as it was assumed that hospitalized women would also be treated in an outpatient setting at some stage.

*JPY*, Japanese yen.
