# Supplementary material for: Economic evaluation of the sFlt-1/PlGF ratio for the short-term prediction of preeclampsia in a Japanese cohort of the PROGNOSIS Asia study
Source: Hypertens Res. 2021 Feb 16;44(7):822–9. doi: 10.1038/s41440-021-00624-2 (PMC8255211; doi:10.1038/s41440-021-00624-2)
Supplement: Supplementary file 3 — Supplementary Figure 1 [file 41440_2021_624_MOESM3_ESM.docx]

**Supplementary Figure 1** Cost savings associated with implementation of the sFlt-1/PlGF ratio in the sensitivity scenario analyses relative to the base-case scenario

**
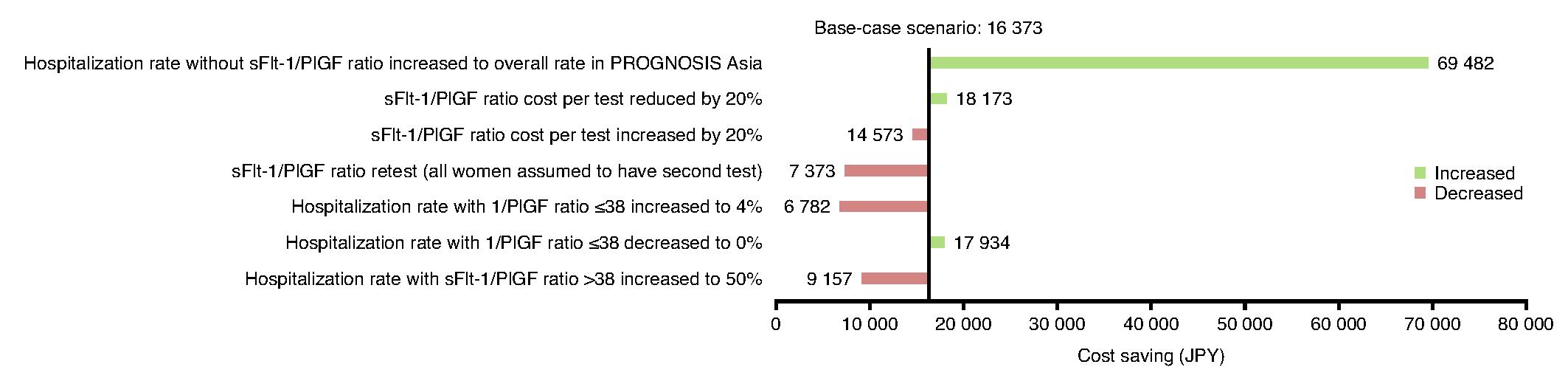
**

*JPY*, Japanese yen; *PlGF*, placental growth factor; *PROGNOSIS*, prediction of short-term outcome in pregnant women with suspected preeclampsIa study; *sFlt-1*, soluble fms-like tyrosine kinase-1.
